# Mutant KRAS in brain endothelial cells promotes vascular inflammation and impairs vascular integrity in brain arteriovenous malformation

**DOI:** 10.1177/0271678X261421424

**Published:** 2026-02-16

**Authors:** Jung-Eun Park, Bridger H Freeman, Hyejin Park, Sehee Kim, Adrian E Bafor, Ohnmar Myint, Song Gao, Zhen Xu, Jakob Körbelin, Jaroslaw Aronowski, Peng Roc Chen, Eunhee Kim, Eun S Park

**Affiliations:** 1 Vivian L. Smith Department of Neurosurgery, McGovern Medical School, The University of Texas Health Science Center at Houston, Houston, TX, USA; 2 Department of Oncology, Hematology and Bone Marrow Transplantation, University Medical Center Hamburg-Eppendorf, Hamburg, Germany; 3 Department of Neurology, McGovern Medical School, The University of Texas Health Science Center at Houston, Houston, TX, USA; 4 Center for Neuroimmunology and Glial Biology, The Brown Foundation Institute of Molecular Medicine, The University of Texas Health Science Center at Houston, Houston, TX, USA

**Keywords:** Brain arteriovenous malformation, KRAS mutation, endothelial cells, microglia, macrophages

## Abstract

Somatic KRAS (KRAS^G12V^) mutation in endothelial cells (EC) induces brain arteriovenous malformation (bAVM) that could lead to vascular instability and ultimately bleeding. However, the causes of bAVM instability remain unclear. Here we demonstrate that KRAS^G12V^ expressing cultured ECs (KRAS-G12V-EC) have increased expression of pro-inflammatory mediators and reduced expression of blood-brain-barrier (BBB) junction constituents. The conditioned medium from KRAS-G12V-EC can activate BV2-microglia (BV2-MG) and conditioned media from this primed BV2-MG can compromise the expression of EC-junction constituents when added to wild-type ECs. In an in vitro BBB model, KRAS-G12V-EC form a dysfunctional EC barrier that is further disrupted, leading to lower transendothelial electrical resistance and increased FITC-dextran leakage when blood-derived macrophages (Mφ) are included. KRAS-G12V-EC potently stimulates BV2-MG chemotaxis. In vitro BBB leakage and BV2-MG chemotaxis were inhibited by treatment with the anti-inflammatory drug minocycline in the KRAS-G12V-EC. In our bAVM mouse model that uses AAV-BR1-KRAS^G12V^ injection to produce EC expression of KRAS^G12V^ in the brain, minocycline injection reduced production of inflammatory cytokines by bAVM nidus, reduced leakage of BSA-647, and restored VE-cadherin expression on malformed vessels. Our findings suggest that KRAS-G12V-EC can activate local MG/Mφ that causes vascular inflammation and instability of malformed vessels in bAVM.

## Introduction

Brain arteriovenous malformations (bAVMs) are a type of cerebrovascular anomalies where arteries connect directly to veins, without an intervening capillary bed.^1^ Although bAVMs are a relatively rare disorder, affecting approximately 18 in 100,000 humans, bAVM patients can have devastating consequences, including permanent neurological impairment or death.^2^ Contemporary treatments for bAVMs include resection, embolization, or radiation to successfully manage the tangled AVM.^3,4^ However, these treatments have varying degrees of success, particularly for bAVMs lying in deep or eloquent brain regions.^3,5^ Therefore, non-operative pharmacological therapies may provide an alternative solution for patients who are poor candidates for invasive interventions.^6^

More than 95% of bAVMs appear as a solitary lesion in patients with no known family history. Although several signaling pathways have been associated with the pathogenesis of bAVMs, recent evidence has implicated somatic mutations of Kirsten rat sarcoma viral oncogene homologue (KRAS) in the majority of sporadic human bAVMs.^7–9^ Mutant KRAS has been explicitly detected in endothelial cells (EC) isolated from bAVM tissues, which preferentially displayed hyperactivation of the mitogen-activated protein kinase (MAPK)/extracellular signal-regulated kinase (ERK) signaling cascade.^7^ This cascade is responsible for regulating multiple transcription factors involved in the regulation of cell survival, migration, differentiation, proliferation, and inflammation.^10,11^ The sufficiency of mutant KRAS to induce development of bAVMs with similar pathogenesis to those in humans has been established with experimental bAVM mice models that use overexpression or activation of mutant KRAS (KRAS^G12V^) in EC.^12^ We termed the model as KRAS^G12V/bEC^ mice.^12^ However, the mechanisms behind how mutant KRAS in EC substantially increase the instability of malformed vessel remains unclear.

More recent evidence indicates that inflammation and immune factors may play a critical role in bAVM pathogenesis. A study comparing ruptured to unruptured human bAVM samples found that the ruptured bAVMs show upregulation in pro-inflammatory genes and downregulation in cell-adhesion genes.^13^ Interestingly, the blood-brain barrier (BBB), which consists of ECs, was found to be leaky in bAVMs, as assessed by dynamic contrast-enhanced MRI (DCE-MRI).^14^ Furthermore, infiltration of macrophages (Mφ) and increased inflammatory cytokines within and around the bAVM lesions have been documented.^13,15^ Activated Mφ may cause vascular degradation and remodeling, in part through the secretion of numerous inflammatory and angiogenic factors, which include TNF-α, IL-6, VEGF, and MMP-9.^16,17^ Recently, single-cell RNA-sequencing confirmed the recruitment of monocytes in bAVMs but not normal tissues.^18^

Microglia (MG), which originate from the embryonic yolk sac, serve as the native myeloid cells within the CNS parenchyma, where they replicate independently of other bone-marrow derived cells.^19,20^ Emerging evidence has been reported that MG interact with BBB components and configure the microenvironment. MG have been shown to stimulate angiogenic vessel sprouting, which promotes the early vessel growth during the developmental stage.^21,22^ MG are activated by constant inflammatory stimuli, which are produced by ECs, causing BBB disruption.^23,24^ Importantly, in bAVM pathology, MG possessing bushy or amoeboid morphologies have been robustly detected in the malformed vessels of mutant KRAS-induced bAVM mice.^12^ MG/Mφ have been shown to contribute to the instability of malformed vessels in various bAVM models, such as the Alk1-deficient HTT mice, in which depletion of MG/Mφ using PLX5622 significantly stabilizes the malformed vasculature.^25^ Still, the role of MG/Mφ in promoting bAVM instability in highly translational mutant KRAS-induced sporadic bAVM is unclear.

Here, we demonstrate that mutant KRAS in EC stimulates the production of inflammatory mediators and causes the loss of junction markers. The KRAS-G12V-modified EC can activate Mφ, which consecutively leads to the loss of EC integrity in an in vitro BBB system. The exacerbation of BBB integrity loss by Mφ and the KRAS-G12V-EC-induced BV2-MG migration, an index of MG activation, is attenuated by treatment of KRAS-G12V-ECs with minocycline. Furthermore, in animal model the KRAS-G12V-EC-activated MG have increased expression of inflammatory mediators. Finally, treating KRAS^G12V/bEC^ mice with minocycline reduced the number of activated/infiltrated MG/Mφ and the production of inflammatory cytokines, and attenuated BBB leakage and loss of a junction protein. In conclusion, our study suggests that KRAS-G12V-EC interacts with MG/Mφ to generate the responses that exacerbate vascular inflammation and leads to the loss of bAVM stability in bAVM mice and that minocycline can inhibit this process.

## Materials and methods

### Animals

All animal care and experimental procedures followed the ethical guidelines outlined in Guide for the Care and Use of Laboratory Animals (Eighth Edition, 2011) from the National Institutes of Health and received approval from the University of Texas Health Science Center at Houston (UTHealth Houston) Center for Laboratory Animal Medicine and Care (CLAMC, AWC-23-0104, IBC-23-048). The Jackson Laboratory (#000664; Bar Harbor, ME, USA) supplied the male and female C57BL/6J mice, which were then bred at CLAMC. The study design and reporting were strictly followed in accordance with the guidelines of ARRIVE and experimental stroke research.^26,27^ For bAVM mouse modeling, age- and sex-matched 6-week-old male and female mice weighing 22–28 g were used. The experimental group was selected at random to minimize bias in accordance with the guidelines of randomized trials,^28^ and the data were analyzed by blinded researchers. The experimental mice were all included in the analysis without exclusion.

### Adeno-associated virus (AAV) production and injection

As we previously reported,^12^ we acquired pXX2-187-NRGTEWD from the University Medical Center Hamburg-Eppendorf. This plasmid contains the adeno-associated virus (AAV)2 rep/cap genes, also known as AAV-BR1 cap, which targets vascular endothelial cells specific to the brain.^29^ A custom AAV production service (Vector Biolabs, Malvern, PA, USA) created AAV-BR1-CAG-human (h) KRAS^G12V^-WPRE and -eGFP-WPRE (as a control) for mouse experiments. Under anesthesia, a retro-orbital (RO) injection of 100 μl of phosphate-buffered saline (PBS) containing 2.5 × 10^10^ genome copies/mouse of AAV-BR1-CAG-hKRAS^G12V^-WPRE or -eGFP-WPRE was given to male and female mice that were 6 weeks old.

### Minocycline treatment

For inhibiting MG/Mφ activation/infiltration and to test the junction protein loss and blood cell leakage, the C57BL6/J mice were treated with minocycline hydrochloride (M9511; Millipore Sigma, Burlington, MA, USA; 10 mg/kg/day, intraperitoneal (i.p.)) or Saline (as a control) for every 2 days, over a period of 4 weeks, starting 2 weeks post-AAV-BR1-KRAS^G12V^ injection. This is the dose determined to be safe in ischemic stroke patients and to reduce infarct size in the rodent ischemic stroke model.^30–33^

### Immunofluorescence staining

Cultured BV2 microglia, macrophages, astrocytes, and endothelial cells were fixed in 10% paraformaldehyde for 20 min before being treated in blocking buffer (1% BSA, 0.3% Triton X-100, 0.02% Tween 20, 10% donkey serum in PBS) for 1 h. The cells were treated with the following primary antibodies overnight and then with the secondary antibodies conjugated to the fluorophore for 1 h at room temperature.

The KRAS^G12V/bEC^ mice were euthanized with isoflurane and were then transcardially perfused with PBS, followed by 10% paraformaldehyde, for tissue fixation. The brains were fixed in 10% formalin at 4°C for 24 h before being immersed in a solution of 30% sucrose and 0.1% sodium azide. The brain, embedded with an optimal cutting temperature compound (OCT, 23-730-571; Fisher Scientific, Hampton, NH, USA), was processed to prepare the 30-μm-thick coronal slices using a cryotome (CM1520; Leika Biosystems, Mannheim, Germany). All sections were heated in citrate buffer at pH 6.0 for 20 min at 80°C before being stained with primary antibodies. The sections were washed three times in cold PBS before being blocked with a blocking solution (1% BSA, 0.3% Triton X-100, 0.02% Tween 20, and 10% donkey serum in PBS) for 1 h at room temperature.

Primary antibodies of goat anti-CD31 (1:200, AF3628; R&D System, Minneapolis, MN, USA), rabbit anti-Iba1 (1:400, 019-19741; Wako, Osaka, Japan), rabbit anti-Ter-119 (1:200, MAB1125; R&D system), rabbit anti-VE-cadherin (1:100, 36-1900; Invitrogen, Waltham, MA, USA), mouse anti-GFAP (1:400, G3893; Sigma-Aldrich, St. Louis, MO, USA), rat ant-F4/80 (1:100, MA1-91124; Invitrogen), mouse anti-KRAS (1:200; Santa Cruz Biotechnology, Dallas, TX, USA), rabbit anti-pERK1/2 (1:500; Cell Signaling Technology, Danvers, MA, USA), mouse anti-IL-1β (1:500, 12242; Cell Signaling Technology), rat anti-IL-6 (1:500, 14-7061-85; Invitrogen), and rabbit anti-caveoline-1 (1:500, 3267; Cell Signaling Technology) were diluted in PBS containing 1% BSA and 0.3% Triton X-100. Secondary antibodies of Alexa 488 conjugated anti-mouse, goat, rabbit, rat (1:500, 715-545-150, 705-545-147, 711-545-152, 712-545-150), Rhodamine Red-X (RRX)-conjugated-anti mouse, goat, rabbit (1:500, 715-295-151, 705-295-147, 711-295-152), and Alexa 647-conjugated-anti mouse, rat (1:500, 715-605-150, 712-605-153), all from Jackson Immunoresearch (West Grove, PA, USA), were diluted in 1 % BSA and 0.3% Triton X-100 in PBS. The non-specific binding of secondary antibodies was tested by omitting primary antibodies. Confocal microscopy (Nikon A1R; Nikon, Japan) was used to analyze the stained sections after they were mounted using clear Fluoromount-G 4′,6-diamidino-2-phenylindole (DAPI) mounting medium (OB010020; SouthernBiotech, Birmingham, AL, USA).

### Image analysis

All quantifications were based on ROIs within bAVM territory, as captured by CD31^+^ expanded vessel morphology. To quantify Ter-119^+^ erythrocytes on immunostained images, these cells were counted by a cell counter from ImageJ using identical ROIs (339.41 μm × 339.41 μm). To measure the mean fluorescence intensity of VE-cadherin, quantification was obtained while masking the endothelial area. To quantify IL-6 and IL-1β expression, confocal images (339.41 μm × 339.41 μm) were analyzed within CD31^+^ endothelial regions or Iba1^+^ MG/Mφ regions. Using ImageJ, binary masks of the CD31^+^ and Iba1^+^ areas were generated and applied to the IL-6 or IL-1β channels to restrict measurements to cell-type-specific compartments. For each ROI, mean fluorescence intensity (MFI; arbitrary units, a.u.) was measured within the masked pixels using the “Measure” function and normalized to the corresponding CD31^+^ or Iba1^+^ area. Image J was used to process the photos, and MATLAB was used to perform quantification calculations using the same ROI from the confocal images (339.41 μm × 339.41 μm).

### In vivo BBB tracer injection

Alexa Fluor 647 conjugate (BSA-Alexa 647; A34785, Invitrogen) was reconstituted in sterile Ringer’s solution to a final concentration of 2.4 mg/ml. At 6 weeks post-AAV-BR1-eGFP or -KRAS^G12V^ injection, the mice received BSA-Alexa 647 (15 mg/kg) intravenously via the tail vein, and the BBB tracer was allowed to circulate for 15 min. Mice were then deeply anesthetized with isoflurane and transcardially perfused with PBS to remove intravascular tracer. The brains were fixed with 10% PFA and processed for immunofluorescence staining as described above. The extravascular area of BSA-Alexa 647 was quantified by measuring the region outside the CD31^+^ vessels using ImageJ.

### Culture of endothelial cell (EC)

BALB/C mouse primary brain microvascular ECs (BALB-5023; Cell Biologics, Chicago, IL, USA) were cultured with a complete medium containing glucose (CM002-050; GenDEPOT, Katy, TX, USA), an EC growth supplement (E2759; Sigma-Aldrich), 10% fetal bovine serum (F0901-050; GenDEPOT), and penicillin-streptomycin (CA005; GenDEPOT) at 37 °C in a 5% CO_2_ humidified incubator. The ECs were cultured at passage five on a flask coated with a type I collagen solution obtained from rat tails (50 μg/ml, C3867; Sigma-Aldrich, Germany). The ECs were seeded on the transwell insert (0.4 μm, 3450, Corning, 2 × 10^5^ cells/transwell) and were then transfected with 1 μg of pAAV-CAG-hKRAS^G12V^ or pAAV-CAG-eGFP (as a control). This transfection agent was created using human KRAS^G12V^ (human KRAS: NM_004985.5), which was inserted into a pAAV transfer plasmid using a lipofectamine transfection reagent (1168027; Invitrogen).

### Culture of astrocytes

C57BL/6 cerebellum astrocytes (C8-D1A, ATCC, CRL-2541) were cultured at passage six in a high glucose Dulbecco’s modified eagle medium (DMEM, ATCC, 30-2002), which included 10% FBS containing 1% penicillin-streptomycin, at 37°C with 5% CO_2_. The astrocytes (1.5 × 10^5^ cells/transwell) were seeded on the basolateral side of the transwell insert (3450; Corning), which was previously seeded with ECs. After 3 h, the EC/astrocyte-seeded transwells were reversed and then co-cultured with or without macrophages.

### Culture of macrophages (Mφ)

Eight weeks old C57BL/6 mice received 2 ml of 3% thiogllycolate (22565;, BD Bioscience, Franklin Lakes, NJ, USA) intraperitoneally to facilitate the population of peritoneal macrophages.^34^ After 2 days, 10 ml of saline was infused into the peritoneal cavity of these mice and the peritoneal macrophages were harvested from this solution. These macrophages cultured on six-well plate (1 × 10^6^ cells/well) with DMEM (CM002-050; GenDEPOT, Katy, TX, USA), including 10 % FBS (F0900-050; GenDEPOT) and 1% P/S (CA005-010; GenDEPOT) and were incubated thereafter for 24 h at 37°C and 5% CO_2_. These macrophages were seeded on 12-well plate (1 × 10^5^ cells/well) to assemble with EC/astrocyte-seeded inserts.

### 3D BBB co-culture system and measurement of transendothelial electrical resistance (TEER)

The EC (luminal side)/astrocyte (Abluminal side)-seeded inserts were used to create a BBB.^35^ The seeded macrophages represent infiltrated macrophages into the brain parenchyma. For the TEER measurements, ECs and astrocytes were fully confluent, allowing for cell-to-cell contact with expression of tight junction proteins. After 24 h, the TEER electrode (EVOM2, World Precision Instrument) was placed in the bottom well and inserted. The TEER measurements (ohm × cm^2^) were performed 24 h before mutant KRAS or GFP transfection and continued daily until the TEER ratio decreased below 50%. The ECs and macrophages were then harvested for RNA analysis, and the ECs, astrocytes, macrophages were used for immunocytochemistry to validate the presence of cells on the 3D BBB co-culture system.

### In vitro vascular permeability assay

The in vitro permeability assay was performed with the collagen I-coated Transwell system (1.0 μm, ECM644; Milipore, Burlington, MA, USA). To construct a BBB and mimic the infiltrated Mφ, EC (luminal side, 1 × 10^5^ cells) and astrocytes (Abluminal side, 5 × 10^4^ cells)-seeded inserts were transferred to the Mφ (1 × 10^5^ cells)-seeded bottom wells in a 24-well plate. The in vitro permeability assay was performed at 72 h after transfection with 1 μg of pAAV-CAG-hKRAS^G12V^ or pAAV-CAG-eGFP (control). Then, inserts were transferred to a fresh receiver plate containing 500 μl of DMEM supplemented with 10% FBS and 1% penicillin-streptomycin in each well, and 150 μl of FITC-dextran (2000 kDa) working solution (1:40 dilution of medium) was added to the upper chamber. After 20 min of incubation at room temperature in the dark, the inserts were removed, and 100 μl of the medium from the lower chamber was collected into a black 96-well microplate for fluorescence measurement. The fluorescence intensity was detected at 485 nm excitation and 535 nm emission using a Synergy HT microplate reader (BioTek, Winooski, VT, USA). Background fluorescence from blank wells was subtracted, and relative permeability was expressed as a percentage of control values. To test the effect of minocycline, the ECs (luminal side) were treated with minocycline (100 μM) or PBS (control) 24 h after transfection with 1 μg of pAAV-CAG-hKRAS^G12V^.

### Microglia (MG) migration assay

BV2 murine MG (CRL-2468; ATCC, Manassas, VA, USA) were cultured at passage four with a medium containing high glucose (CM002-050; GenDEPOT, Katy, TX, USA), 10% fetal bovine serum (F0901-050; GenDEPOT), and penicillin-streptomycin (CA005; GenDEPOT) at 37°C in a 5% CO_2_ humidified incubator. For the BV2-MG migration assay, BV2 cells were cultured on transwell inserts (8 μm, 3422; Corning, St. Louis, MO, USA). ECs were seeded on a 12-mm coated coverslip (50-143-822; Fisher Scientific, Hampton, NH, USA), which was positioned on a 24-well chamber. ECs were then transfected with pAAV-CAG-hKRAS^G12V^ or pAAV-CAG-eGFP-WPRE (as a control) using a lipofectamine transfection reagent (1168027; Invitrogen). After 24 h, the BV2-seeded transwell inserts were transferred to the EC-seeded 24-well chamber and incubated for 12 h. In the minocycline treatment experiment, minocycline hydrochloride (100 μM, M9511; Millipore Sigma, Burlington, MA, USA) or PBS (as a control) was administered to the EC 1 day after transfection with mutant KRAS or GFP, before combining with BV2-seeded transwell inserts. The EC and BV2 cells were immunostained with CD31 and Iba1 Antibodies (primary) and goat-488 and rabbit-RRX (secondary) 4 days after combining the BV2-seeded transwell and EC-seeded 24-well chamber. Images were then captured using a Nikon A1R with 20X zoom.

### Secretome analysis of in vitro cytokine, chemokine, angiogenic factor assays

The conditioned medium (CM) samples were harvested from KRAS-G12V-EC or GFP-EC (insert) or macrophages (bottom well) in 3D BBB co-culture system. Cytokine, chemokine and angiogenic factors were measured using a Proteome Profiler Mouse Chemokine Array Kit 1 (ARY020; R&D Systems, Minneapolis, MN, USA), Proteome Profiler Mouse Cytokine Array Kit Panel A (ARY006; R&D Systems), and Proteome Profiler Mouse Angiogenesis Array Kit (ARY015; R&D Systems). The secretome assay was performed according to the manufacturer’s instructions (R&D Systems). Briefly, each CM was mixed with a cocktail of biotinylated detection antibodies and incubated with each array membrane. The capture antibodies were bound to specific target proteins, which are spotted in duplicate. Captured proteins are visualized using chemiluminescent detection reagents. The visualized spots were analyzed using the software ImageJ.

### Transfer of conditioned medium

The CMs were harvested from ECs transfected with p.AAV-KRAS^G12V^ or p.AAV-eGFP at 3 days. The CMs were centrifuged at 300 × *g* for 5 min, and the supernatants were added to the other cells at a 1:1 volume ratio with each cell type’s complete growth medium. The KRAS-EC- or GFP-EC-CM were transferred to the BV2-MG or directly to the normal ECs. After 48 h of incubation with BV2-MG, the BV2-CM were further transferred to the normal ECs. At each step, the KRAS-EC- or GFP-EC-CM-treated BV2-MG ((b) in Figure 4), BV2-MG-CM-treated normal ECs ((c) in Figure 4), or KRAS-EC- or GFP-EC-CM-treated normal ECs ((d) in Figure 4) are harvested and analyzed for the mRNA level changes in inflammatory mediators or EC junctions.

### Western blot

The ECs transfected with pAAV-CAG-hKRAS^G12V^ or pAAV-CAG-GFP were lysed using a protein lysis buffer (GenDEPOT, 50 mM Tris–HCl, pH 7.5, 150 mM NaCl, 1% triton X-100, 2 mM EDTA, 1% sodium deoxycholate, 0.1% SDS) containing a Phosphatase Inhibitor Mixture (P3200-005; GenDEPOT) and Protease Inhibitor Mixture (P3100-005; GenDEPOT). The lysate was centrifuged at 14,000 rpm for 20 min, and the supernatant was collected and used for western blot. Proteins were quantified using BCA kit (P8100-050; GenDEPOT). Proteins (25 µg) were boiled in a loading buffer (L1100-001; GenDEPOT) and separated by 4-20% ExpressPlus™ Page Gel (M42015; GenScript, Piscataway, NJ, USA) using a Mini-PROTEAN Tetra Cell (1658004; Bio-Rad Laboratories, Hercules, CA, USA). After electrophoresis, the proteins were transferred to polyvinylidene difluoride membranes (1704273; Bio-Rad Laboratories) using the Trans-Blot Turbo Transfer System (1704150; Bio-Rad Laboratories). The following primary antibodies were used in this study: GFP (1:1,000, 2956; Cell Signaling Technology), KRAS^G12V^ (1:1,000, MA5-42375; Invitrogen), p-ERK (1:1,000, 9101; Cell Signaling Technology), ERK (1:1,000, 9102, Cell Signaling Technology), and β-actin (1:1,000, sc-47778; Santa Cruz Biotechnology). The next day, the blots were incubated with secondary antibodies: Rabbit IgG Horseradish Peroxidase-conjugated antibody (1:2,000, HAF008; R&D Systems) or Mouse IgG Horseradish Peroxidase-conjugated antibody (1:2,000, HAF007; R&D Systems).

### Real-time quantitative PCR

Total mRNA was extracted from mouse cell cultures using an RNA extraction kit (PURY RNA Plus, P2030-050; GenDepot) for cell cultures according to manufacturer’s protocols. cDNA was synthesized using a commercial kit (High-Capacity cDNA Reverse Transcription Kits, 4368814; Applied Biosystems, Foster City, CA, USA) and the mRNA expression levels were measured by Real-Time qRT-PCR reaction (Applied Biosystems Quant 3 Studio qPCR) using amfisure qGreen master mix (Q5603-005; GenDEPOT). PCR primers were purchased from Sigma-Aldrich. The following primers were used: KRAS^G12V^: Forward (F)-5′AGGCCTGCTGAAAATGACTGAATAT, Reverse (R)-5′GCTGTATCGTCAAGGCACTCTT, IL-1β: F-5′CAACCAACAAGTGATATTCTCCATG, R-5′GATCCACACTCTCCAGCTCGCA, IL-6: F-5′TGGTACTCCAGAAGACCAGAGG, R-5′AACGATGATGCACTTGCAGA, MCP-1: F-5′GCATCCACGTGTTGGCTCA, R-5′CTCCAGCCTACTCATTGGGATCA, VEGF-A: F-5′CTCACCAAAGCCAGCACATA, R-5′AATGCTTTCTCCGCTCTGAA, CDH5: F-5′TAGCAAGAGTGCGCTGGAGATTCA, R-5′ACACATCPTAGCTGGTGGTGTCCA, TJP1: F- 5′GATTTACCCGTCAGCCCTTCT, R-5′TCGCAAACCCACACTATCTCC, OCLN: F-5′AGATTCCTCTGACCTTGAGTGTGG, R-5′TCCTGCTTTCCCCTTCGTG, CLDN5: F-5′CCAGAGCAGAGGCACCAGA, R-5′AGACACAGCACCAGACCCAGA, TNF-α: F-5′ATGGCCTCCCTCTCATCAGT, R-5′TTTGCTACGACGTGGGCTAC, MMP-9: F-5′GCCGACTTTTGTGGTCTTCC, R-5′TACAAGTATGCCTCTGCCAGC, CSF1R: F-5′TGAGCAAGACCTGGACAAGGA, R-5′CCGCTGGTCAACAGCACGTTT. The data was analyzed by the comparative CT method (ΔΔCT method; Applied Biosystems Quant 3 Studio qPCR).

### Statistical analysis

Statistical analyses were performed using GraphPad Prism 10 (GraphPad Software, San Diego, CA, USA). Data are presented as mean ± standard deviation (SD) unless otherwise stated. Statistical significance was determined by unpaired student’s *t*-test for comparisons between two experimental groups. One-way analysis of variance (ANOVA) with Tukey’s multiple comparisons test was performed in multiple groups. Differences were considered significant at *p* < 0.05.

## Results

### Mutant KRAS induces vascular inflammation in bAVM mice

**Figure 1.**
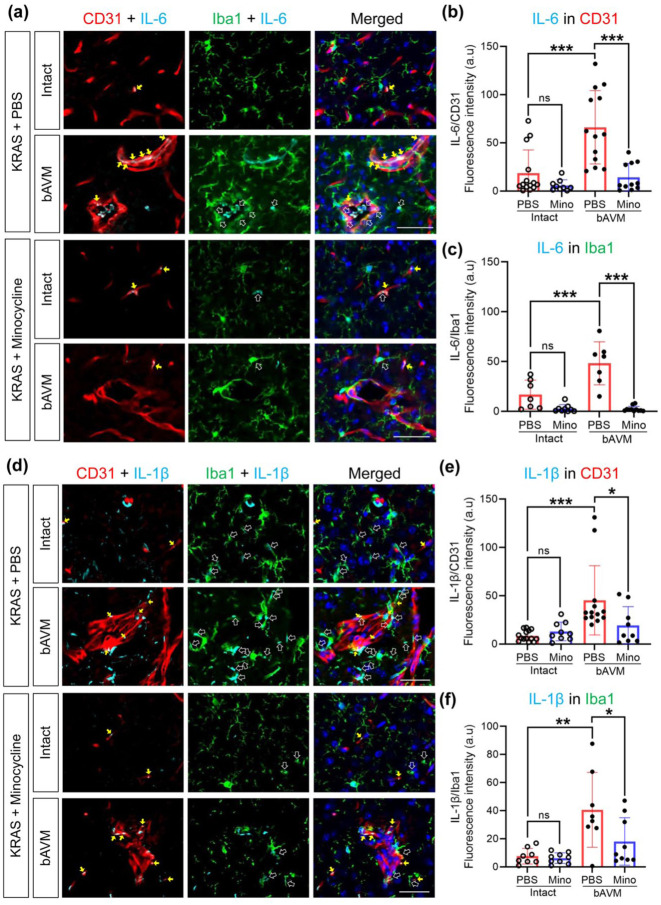
Treatment of KRAS^G12V/bEC^ mice with minocycline reduces IL-6 and IL-1β expression on the EC and MG/Mφ. (a, d) Representative images of immunofluorescence showing the expression of IL-6 (a) and IL-1β (d) on the CD31^+^ and Iba1^+^ cells by AAV-BR1-KRAS^G12V^ injection with or without treatment with minocycline; The yellow arrow indicates expression of IL-6 or IL-1β in CD31^+^ vessels, while white empty arrow indicates expression of IL-6 or IL-1β in Iba1^+^ MG/Mφ; minocycline decreased IL-6 and IL-1β expression on the CD31^+^ and Iba1^+^ cells in bAVM area. Scale bar = 50 μm; (b, c, e, and f) Bar graphs quantifying the intensity of IL-6 (b, c) or IL-1β (e, f) expression in area (μm^2^) of CD31^+^ vessels (b, e) or Iba1^+^ MG/Mφ (c, f). The quantifications (arbitrary units, a.u) are normalized as the ratio of IL-6 (or IL-1β) intensity to the Iba1^+^ or CD31^+^ area. Each dot indicates a randomly selected ROI (*n* = 7–9) obtained from mice (*n* = 4–6). ANOVA with Turkey’s multiple comparisons test. *p < 0.05. **p < 0.01. ***p < 0.001.

We earlier characterized our novel bAVM mouse model, based on overexpression of the somatic mutant variant KRAS^G12V^.^12^ We injected an AAV-BR1-KRAS^G12V^, which induces expression of KRAS^G12V^ specifically in the brain endothelial cells (ECs). We termed the model as KRAS^G12V/bEC^ mice. The model displays the CD31 (immunopositive, “+”) dysplastic vessels and robust presence of Iba1^+^ microglia (MG) and macrophages (Mφ) 6 weeks post-AAV-BR1-KRAS^G12V^ injection (Figure 1(a) and (d)). In the earlier analysis of bAVM tissues, we observed a bushy or amoeboid morphology of MG (or Mφ) and elevated mRNA levels of inflammatory mediators, including IL-6, IL-1β, TNF-α, MMP-2, and MMP-9, in the bAVM area compared to the intact area.^12^ Using antibodies for representative inflammatory cytokines IL-6 and IL-1β, we now performed immunofluorescence staining. We determined that Iba1^+^ MG/Mφ indeed express IL-6 and IL-1β in the bAVM area compared to intact brain in KRAS^G12V/bEC^ mice (Figure 1(a), (c), (d), and (f), Supplemental Figures 1 and 2). In parallel, we established that ECs in the bAVM also produce IL-6 and IL-1β (Figure 1(a), (b), (d), and (e); Supplemental Figures 1 and 2). These data indicate that ECs-carrying mutant KRAS exacerbate vascular inflammation by inducing inflammatory cytokines in both MG/Mφ and ECs in KRAS^G12V/bEC^ mice.

Regarding the vascular inflammation, we next tested if the anti-inflammatory strategies reduce the vascular inflammation in the bAVM area. Minocycline, an FDA-approved antibiotic, has been used for reducing inflammation, neuroprotection, and treating acute ischemic and hemorrhagic stroke.^30,36–38^ Thus, to test the therapeutic relevance of minocycline, we next treated the bAVM mice with minocycline starting 2 weeks post-AAV-BR1-KRAS^G12V^ injection. Four weeks later, we showed that minocycline effectively reduced the expression of IL-6 and IL-1β in CD31^+^ ECs and Iba1^+^ MG/Mφ in the bAVM area (Figure 1(a)–(f)). The data suggest that minocycline modulates the vascular inflammation in bAVM mice. Still, the mechanism by which the EC KRAS mutation causes vascular inflammation is unclear.

### KRAS^G12V^ overexpression promoted inflammation and loss of integrity in EC

**Figure 2.**
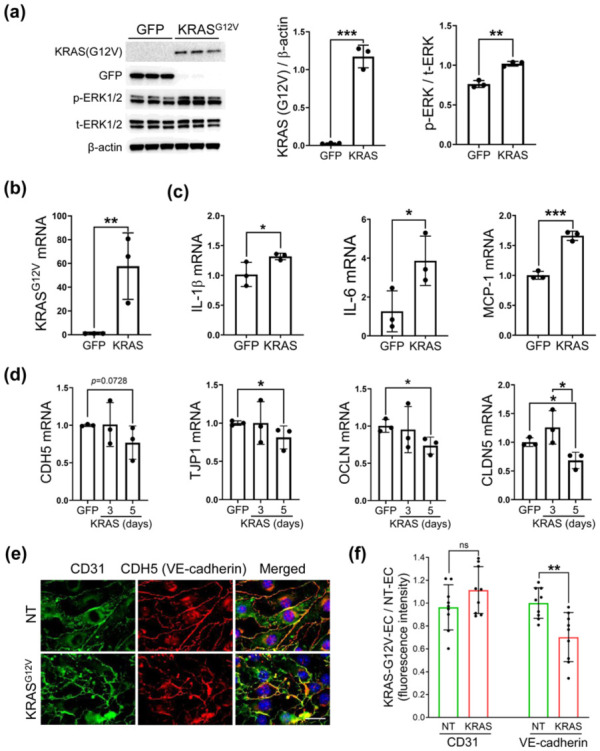
Activating KRAS^G12V^ alters EC homeostasis in vitro. (a) Western blot images and bar graphs showing the increased KRASG12V and p-ERK1/2 proteins 3 days after cultured mouse EC transfection with KRASG12V compared to GFP; (b) Bar graphs showing overexpression of KRASG12V mRNA at 3 days after KRASG12V transfection compared to GFP on cultured mouse ECs; (c) Bar graphs showing increase of inflammatory cytokines (IL-1β, IL-6), and chemokines (MCP-1) in KRAS-G12V-EC at 3 days; (d) Bar graphs quantifying decrease of mRNA levels in EC junction markers, CDH5, TJP1, OCLN, and CLDN5 5 days after KRASG12V transfection compared to GFP on cultured mouse ECs; (e, f) Representative immunofluorescence images and bar graphs showing reduced CDH5 (VE-cadherin) expression without loss of CD31 expression on cultured mouse ECs at 5 days after KRASG12V transfection compared to NT (non-transfected). Scale bar = 20 μm. Unpaired *t*-test. All experiments are repeated three times. ns indicates no significant differences. *p < 0.05. **p < 0.01. ***p < 0.001.

Mutant KRAS is a well-known trigger for tumor-promoting inflammation.^39–41^ However, this effect has yet to be verified for bAVM ECs. Confirming this effect would provide additional evidence that mutant KRAS in ECs acts as trigger of inflammation in the bAVM area. Therefore, we analyzed the expression of inflammatory mediators in mutant KRAS-transfected mouse ECs (KRAS-G12V-EC) cultured in vitro. Specifically, cultured mouse ECs were transfected with a plasmid containing KRAS^G12V^ or GFP (control). At 3 days, we observed increased mutant KRAS^G12V^-specific proteins and mRNAs, and phospho-(p)-ERK1/2, downstream of activating KRAS, in KRAS^G12V^-transfected ECs compared to the control (Figure 2(a) and (b)). In the cultures, KRAS^G12V^ increased the mRNA expression of the pro-inflammatory cytokines (IL-1β and IL-6), as well as monocyte chemoattractant protein-1 (MCP-1), a chemokine that can regulate Mφ attraction, as measured 3 days after KRAS^G12V^ transfection compared to GFP (Figure 2(c)). Next, we examined the effect of KRAS^G12V^ on the mRNA expression of BBB-associated junction molecules in ECs, using qPCR and immunofluorescence staining. The mRNA levels of adherens junction molecules (CDH5/VE-cadherin) and tight junction molecules (TJP1, OCLN, and CLDN5) were significantly downregulated in ECs carrying KRAS^G12V^ 5 days after KRAS^G12V^ transfection compared to GFP in cultured mouse ECs (Figure 2(d)). In parallel, KRAS-G12V-EC displayed the lower expression of CDH5 (VE-cadherin) compared to the non-transfected (NT) ECs (Figure 2(e) and (f)). These results indicate that the KRAS^G12V^ mutation induces a loss of EC homeostasis by increasing inflammatory cytokine and chemokine production, and this mutation likely drives a loss in BBB integrity.

### Macrophages orchestrate the breakdown of the EC barrier integrity

**Figure 3.**
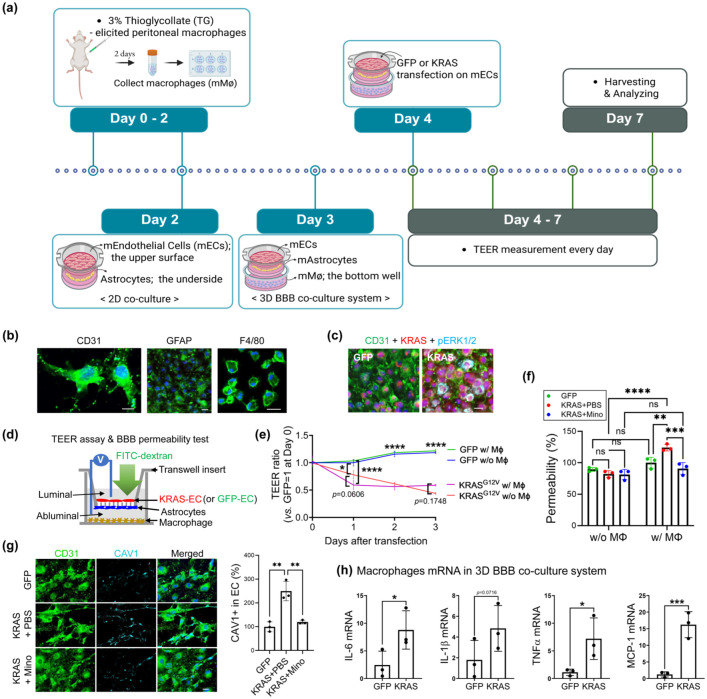
Mφ enhance the BBB disruption in a 3D BBB co-culture system. (a) A schematic diagram of the 3D BBB co-culture system (see details at section “Materials and Methods”). The scheme was created with Biorender.com; (b) Immunofluorescence staining showing cultured ECs (CD31), astrocytes (GFAP), and Mφ (F4/80) on the abluminal side of the insert and coverslip in the culture plate. Scale bar = 20 μm; (c) Immunofluorescence staining showing ERK1/2 phosphorylation in ECs by KRAS mutation, compared to GFP. Scale bar = 20 μm; (d, e) Transendothelial electrical resistance (TEER) assay showing mutant KRAS reduced EC barrier resistance in the co-cultured system with EC, Astrocytes, and with or without Mφ starting 1-day post-KRAS^G12V^ transfection. Higher electrical resistance indicates that the EC junctions are intact, indicating that the mimicked BBB structure has not been compromised. Note that the co-culture with Mφ showed significantly reduced EC barrier resistance at 1-day post-combination of the transwell with the bottom well. Data are presented as mean ± standard error of the mean (SEM); (d, f) In vitro BBB permeability test showing that the permeability of FITC-dextran is unchanged in KRAS-EC w/o Mφ at 3-day post-KRAS^G12V^ transfection, whereas it is significantly increased in the co-culture with Mφ. Note that the treatment with minocycline (Mino, 100 μM, at 24 h after transfection with KRAS^G12V^) to the ECs in the luminal side showed significantly reduced permeability; (g) Representative immunofluorescence images and bar graphs showing increased Caveolin-1 (CAV1) expression on cultured mouse ECs at 3-day post-KRASG12V transfection compared to GFP (control) in a 3D BBB co-culture system. Note that the treatment with minocycline (Mino) to the ECs showed a significant reduction of CAV1 expression in ECs. Scale bar = 20 μm. (h) Bar graphs showing increased mRNA levels of inflammatory cytokines (IL-6, IL-1β, and TNF-α) and chemokine MCP-1 in Mφ were observed in the 3D BBB co-culture system. ANOVA or Unpaired *t*-test. *p < 0.05. **p < 0.01﻿. ***p < 0.001. ****p < 0.0001.

The robust presence of activated MG and infiltrated Mφ has been reported in the human bAVM tissues and previously reported in our bAVM mouse model.^12,15,18^ Additionally, extensive vascular inflammation caused by the interaction between ECs and myeloid cells has also been reported.^42,43^ However, the mechanisms by which infiltrated Mφ-induced inflammation causes the loss of vascular integrity in bAVM territory are unknown. We established a 3D BBB mouse co-culture system by seeding mutant KRAS-G12V-ECs on the upper surface of the transwell membrane, astrocytes (GFAP^+^) on the underside of the membrane, and Mφ (F4/80^+^) in the bottom wells (to mimic Mφ, infiltrated into the parenchymal side of the BBB) (Figure 3(a) and (b)). Three days after the transfection of GFP (control) or mutated KRAS into the ECs, we confirmed enhanced phosphorylation of ERK1/2 in KRAS-G12V-EC (Figure 3(c)), which was consistent with prior reports.^7,12^

We further used the TEER assay to daily evaluate the integrity of the BBB structure following transfection of ECs with GFP or KRAS^G12V^. The lower electric resistance measured by TEER assay indicates the loss of EC integrity.^44^ Here, the lower TEER ratio was already detected on day 1 post-transfection in an in vitro 3D BBB model, suggesting that mutant KRAS acts as trigger leading to the loss of EC integrity (Figure 3(d) and (e)). Notably, presence of Mφ significantly enhanced the reduction of the TEER ratio up to 42.6% (*p* < 0.001) at day 1 post-transfection compared to the BBB co-cultures without Mφ (only 21.9%, *p* < 0.05) (Figure 3(e)). Interestingly, despite a reduction in the TEER ratio, FITC-dextran, a BBB tracer, does not pass through the transwell insert on day 3 post-transfection (Figure 3(f)). However, the presence of Mφ significantly enhanced FITC-dextran permeability in a 3D BBB coculture system. To next test whether the inflamed KRAS-G12V-EC initiated an increase in BBB permeability, we treated ECs (luminal side) with minocycline (100 μM) 24 h after transfection with pAAV-CAG-hKRAS^G12V^. After 48 h, we measured fluorescence intensity of the medium on the abluminal side and, notably, minocycline treatment in the KRAS-G12V-EC on day 1 post-transfection markedly reduced the increased BBB permeability (Figure 3(f)). We next tested changes in caveolin-1 (CAV1) expression, which mediates transcytosis across the BBB.^45^ The KRAS-EC led to increased CAV1 expression, whereas minocycline treatment reduced CAV1 expression in ECs (Figure 3(g)). Finally, we used real-time PCR to examine the effects of KRAS-G12V-ECs on Mφ in a 3D BBB co-culture system. KRAS-G12V-EC stimulated IL-6, IL-1β, TNFα, and MCP-1 mRNA expression in the lower chamber Mφ (Figure 3(f)). These findings suggest that KRAS-G12V-ECs alone can compromise the BBB integrity, and the interaction between KRAS-G12V-ECs and inflammatory Mφ may significantly exacerbate the impairment of BBB integrity.

### Mutant KRAS induces the release of chemokines, cytokines, and angiogenic factors from KRAS-G12V-ECs and activates Mφ

Next, we analyzed the secretome in the conditioned medium (CM) of KRAS-G12V-EC-CM (in the upper chamber in the 3D BBB co-culture system; Figure 3(d)), to establish whether mutant KRAS increases the expression and release of Mφ attracting factors. Dot blot images compared the release of chemokines, inflammatory cytokines, and angiogenic molecules in ECs transfected with GFP versus KRAS^G12V^. We observed an increased production of chemokines like MCP-1, inflammatory molecules like IFN-γ, IL-1α, IL-16, IL-1β, IL-17, and IL-23, and angiogenic molecules like MIP-1α, MMP-9, angiopoietin-1,3, IGFBP-1,2,3, and endoglin (Supplemental Figure 3(a) and (b)). These findings suggest that mutant KRAS-G12V-ECs produce humoral mediators, which may stimulate immune response of adjacent Mφ. This evidence supports our hypothesis that KRAS-G12V-ECs may recruit Mφ and activate inflammation in the malformed vasculature of bAVMs.

Thus, we next performed a corresponding quantification of the released chemokines, cytokines, and angiogenic factors in the lower chamber of the 3D BBB co-culture system (Figure 3(d)) containing Mφ-CM (Supplemental Figure 3(c) and (d)). The secretome analysis of Mφ-CM revealed a significant increase in the levels of chemokines (SDF-1, C5/C5a, CCL28), cytokines (IL-10), and angiogenic molecules (MMP-3 and MMP-9) (Supplemental Figure 3(c) and (d)). These findings support the possibility that the activated Mφ at the site of bAVM through their secretome can compromise the EC barrier integrity, as we demonstrated through TEER assay in the BBB co-culture system in the presence of KRAS-G12V-ECs-activated Mφ (Figure 3(f)).

### KRAS-G12V-ECs induced recruitment and activation of MG

**Figure 4.**
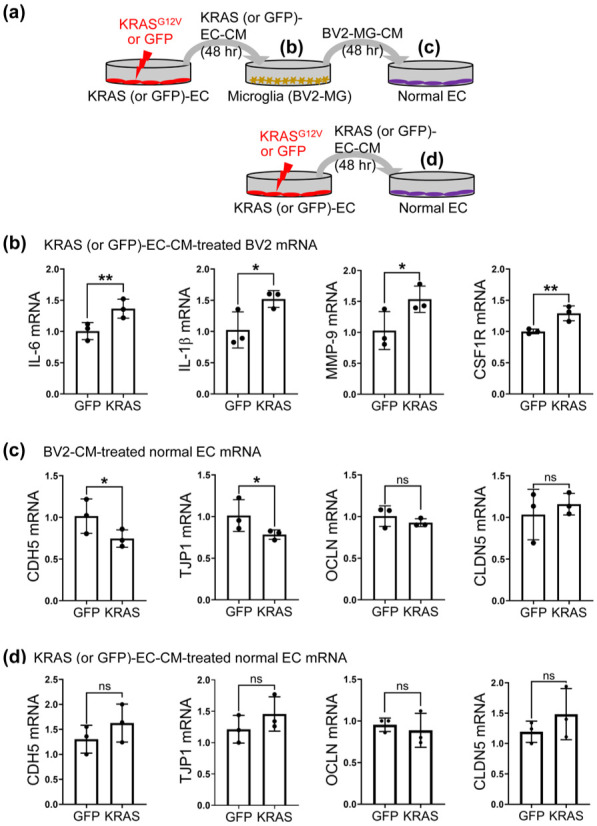
MG corroborate with KRAS-G12V-EC to promote the loss of EC integrity in vitro. (a) A schematic diagram of the experiment. KRAS-G12V-EC-CM (conditioned medium) was added to BV2-MG and incubated for 48 h (*for b*), and then BV2-CM was transferred to control EC for 48 h (*for c*), or KRAS-G12V-EC-CM was added to control EC for 48 hours (*for d*); (b) Bar graphs showing increased mRNA levels in inflammatory cytokines (IL-6, IL-1β), proteolytic enzymes (MMP-9), and the MG-survival factor (CSF1R) in KRAS-G12V-EC-CM treated BV2-MG; (c) Bar graphs showing decreased mRNA levels of junction markers, CDH5 and TJP1, in EC after 2 days of incubation with BV2-CM, while OCLN and CLDN5 did not change; (d) Bar graphs showing non-significant changes in mRNA levels of junction markers in EC after 2 days of incubation with KRAS-G12V-EC-CM. Unpaired *t*-test. ns indicates no significant differences. All experiments are repeated three times. *p < 0.05. **p < 0.01.

We previously observed that Iba1-immunostaining exhibits the bushy or amoeboid morphology of activated MG in the bAVM area.^12^ Therefore, to examine how KRAS-G12V-ECs interact with MG, we treated BV2-MG with KRAS-G12V-EC-CM and 48 h later we analyzed transcriptome changes in BV2-MG (Figure 4(a)). We observed that BV2-MG treated with KRAS-G12V-EC-CM have increased mRNA expression of inflammatory cytokines (*IL-6* and *IL-1β*), BBB breakdown-related enzymes (*MMP-9*), and MG-development/proliferation-related receptor (*CSF1R*) (Figure 4(b)). Next, we treated normal ECs with the BV2-MG-CM to examine how activated BV2-MG influence normal ECs. Normal ECs treated with BV2-MG-CM had decreased mRNA expression of cell adhesion molecules (*CDH5* and *TJP1*) after 48 h of incubation (Figure 4(c)). However, normal ECs treated with KRAS-G12V-EC-CM had no changes in the mRNA expression of cell junction molecules compared to the GFP-EC-CM-treated normal ECs (Figure 4(d)).

**Figure 5.**
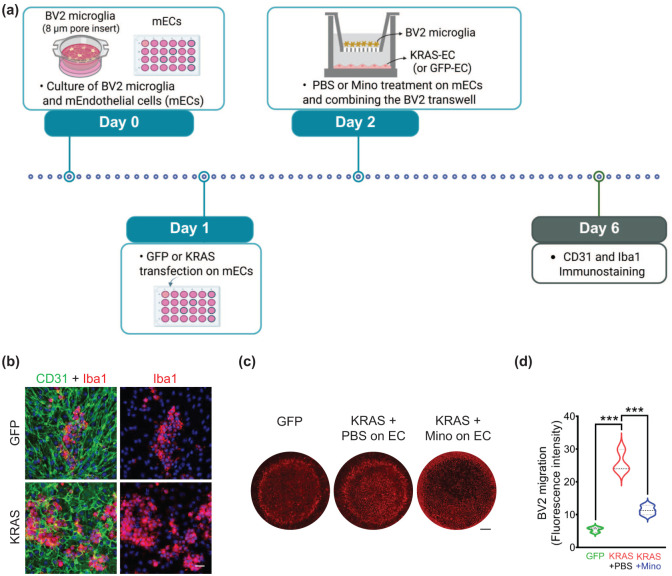
Minocycline attenuates KRAS-G12V-EC-induced MG migration in vitro. (a) BV2-MG transmigration activities were tested using a transwell insert (please see the detailed procedure in the section “Materials and methods”); (b) Immunofluorescence staining showing accelerated BV2-MG migration 12 h later by KRAS mutation in ECs. Scale bars = 20 μm; (c, d) Minocycline (100 μM) or PBS was treated on ECs 1 day post-mutant KRAS (or GFP) transfection to test the effects on BV2-MG migration. Bar graphs showing migrated Iba1^+^ MG in the bottom well at 4 days post-minocycline treatment, compared to PBS. ANOVA with Turkey’s multiple comparisons test. Scale bars = 100 μm. ***p < 0.001.

In response to injury, inflammatory MG can migrate to the injured site.^46,47^ However, it is unknown if MG migrate toward the mutated EC. To address the migration responses, we used transwell migration assay and co-cultured EC-transfected with GFP or KRAS^G12V^ and BV2-MG (Figure 5(a)). BV2-MG were seeded on the upper side of the transwell membrane, and GFP-ECs or KRAS-G12V-ECs were in the bottom wells. Immunofluorescence staining revealed that mutant KRAS potently enhanced BV2-MG migration to the bottom wells of KRAS-G12V-ECs, compared to the GFP-ECs, as measured at 12 h after co-cultures (Figure 5(b)).

MG activation and migration depend on inflammatory or chemotactic stimuli derived from ECs that produce a diverse range of cytokines and chemokines.^46,48^ The expression of such inflammatory mediators can be suppressed by minocycline.^49,50^ Therefore, to test whether the suppression of inflammatory responses in ECs modulate MG migration, we treated KRAS-G12V-EC or GFP-EC with minocycline before BV2-MG-transwell transfer to the EC-seeded chamber. We found that minocycline almost completely reversed the migration enhancement toward KRAS-G12V-EC (Figure 5(c) and (d)). These findings suggest that targeting the inflammatory response instigated by KRAS-G12V-ECs may be a promising strategy for modulating the presence of pro-inflammatory MG at the malformed vessels of bAVM.

### Treatment with minocycline attenuates the bAVM instability

**Figure 6.**
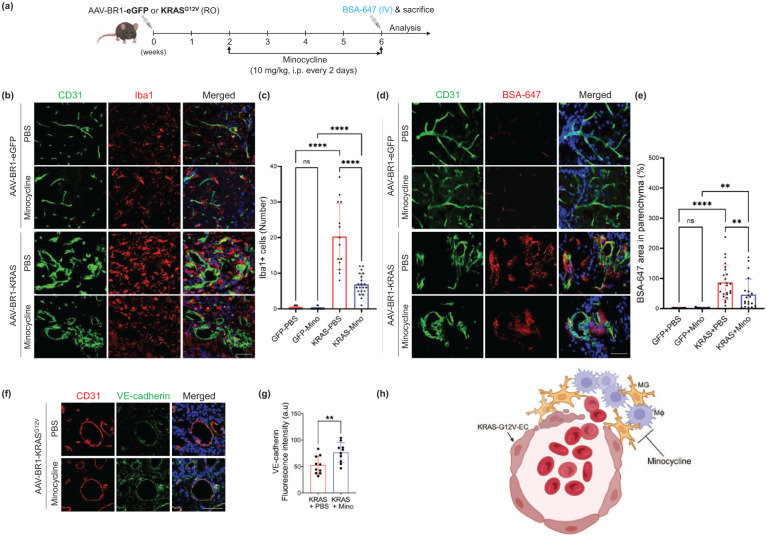
Minocycline treatment enhances the BBB integrity in KRAS^G12V/bEC^ mice. (a) KRAS^G12V/bEC^ mice received minocycline every 2 days (i.p., 10 mg/kg) starting 2 weeks post-AAV-BR1-KRASG12V or -eGFP injection for 4 weeks; (b) Iba1 immunostaining shows a decrease in the numbers of MG/Mφ surrounding bAVM following minocycline (Mino) treatment in KRASG12V/bEC mice; Scale bar = 50 μm; (c) Bar graphs quantifying the number of Iba1+ cells. ANOVA with Turkey’s multiple comparisons test.. Each dot indicates a randomly selected ROI (*n* = 14–21) obtained from the mice (*n* = 5 for GFP-PBS and KRAS-PBS, or *n* = 10 for GFP-Mino and KRAS-Mino); (d) Representative immunofluorescence image shows a leakage of BSA-647 in KRASG12V/bEC mice. Notably, treatment with minocycline (Mino) to KRASG12V/bEC mice attenuated leakage of BSA-647; Scale bar = 50 μm; (e) Bar graphs quantifying the area of BSA-647 leakage in parenchyma. ANOVA with Turkey’s multiple comparisons test. Each dot indicates a randomly selected ROI (*n* = 17–24) obtained from the mice (*n* = 5 for GFP-PBS and KRAS-PBS, or *n* = 10 for GFP-Mino and KRAS-Mino); (f) Immunofluorescence staining showing VE-cadherin (Cdh5) restoration on the CD31+ bAVM vessels; Scale bar = 50 μm; (g) Bar graphs quantifying VE-cadherin expression in CD31+ vessels. Unpaired *t*-test. Each dot indicates a randomly selected ROI (*n* = 10) obtained from mice (*n* = 5); (h) Minocycline treatment attenuates BBB leakage by targeting inflammatory cells, thereby increasing bAVM stability in mice. **p < 0.01. ****p < 0.0001.

The above in vitro findings with minocycline are well corroborated by the results of our locally established bAVM mouse model. We showed that KRAS^G12V/bEC^ mouse treated with minocycline (10 mg/kg, i.p.) for 4 weeks starting 2 weeks post-transfection, as compared to saline (control for minocycline) or AAV-BR1-eGFP-injected mice (control for KRAS^G12V^; Figure 6(a)), demonstrated reduced number of Iba1^+^ MG/Mφ in the bAVM mouse (Figure 6(b) and (c)). Notably, the leaking of BSA-647, a BBB tracer, in the KRAS^G12V/bEC^ mouse was significantly attenuated by minocycline treatment (Figure 6(d) and (e)), which is recapitulated by the attenuated infiltration of Ter-119^+^ red blood cells (Supplemental Figure 4). In correspondence to the attenuated leaking of BBB tracer, the increased expression of vascular endothelial (VE)-cadherin, a junction protein, was observed in the CD31^+^ bAVM vessels in minocycline-treated KRAS^G12V/bEC^ mice (Figure 6(f) and (g)). These data suggest that minocycline significantly inhibited MG/Mφ activation/recruitment toward the bAVM area and improved bAVM integrity in the KRAS^G12V/bEC^ mice (Figure 6(h)).

## Discussion

The research on bAVM has shown that the malformed vessels of bAVMs are prone to rupture, potentially leading to intracerebral hemorrhage (ICH).^51–55^ Treatments for bAVMs are commonly directed toward mitigating urgent cases of ruptured bAVM, rather than targeting the risk factors that weaken malformed vessels at high risk of bAVM rupture. The present study demonstrates that the instability of bAVM is due to vascular inflammation that contributes to the weakening of malformed vessels. Specifically, we show that the KRAS^G12V^ mutation in EC drives an inflammatory response in EC, attracting inflammatory MG/Mφ to the bAVM in KRAS^G12V/bEC^ mice. The sequelae and underlying mechanisms were rigorously validated using a cultured BBB system, which clearly demonstrated that KRAS-G12V-EC induce pro-inflammatory responses and disrupt BBB integrity, especially in the presence of co-cultured MG/Mφ. This suggests that the pro-inflammatory phenotype of KRAS-G12V-EC, through its secretome, induces the recruitment and activation of MG/Mφ, which further amplifies vascular inflammation and causes instability in bAVM vessels.

Despite recognition of diverse neurovascular cells involved in bAVM pathology, it remains unclear why these malformed vessels weaken. ECs play a critical role in the formation and maintenance of the BBB. In comparison to other organs, brain ECs produce substantially higher levels of junctional adhesion molecules like CDH5, TJP1, OCLN, and CLDN5, which function to regulate permeability and increase cohesion among cells comprising the BBB.^56,57^ This resilient and semi-permeable barrier is essential for brain homeostasis and vascular integrity. However, as we demonstrated in this study, the mutant KRAS^G12V^ induced a MEK/ERK-mediated inflammatory response in ECs, leading to a loss of junction markers and potentially resulting in BBB disruption. In particular, the KRAS-EC-CM-stimulated BV2-MG-CM reduced CDH5 and TJP1 in ECs. Regarding the earlier reduction of CDH5 and TJP1 than OCLN and CLDN5 in response to inflammatory stimuli, the selective downregulation of CDH5 and TJP1 may reflect the initial impairment of BBB integrity.^58–61^ Since disruption of the BBB compromises the structural integrity of malformed vessels in mice, the findings we presented here suggest that the inflammation originating from KRAS-G12V-ECs directly contributes to the instability of malformed vessels in bAVM. Meanwhile, BV2-MG are commonly used for testing neuroinflammation, however, BV2-MG may not fully reflect in vivo microglial in several aspects, including faster proliferation, higher baseline activation, increased production of inflammatory cytokines, and altered transcriptional profiles.^62–65^ Thus, the test with BV2-MG has a limited ability to recapitule the in vivo mechanisms of MG involvement in BBB disruption and EC barrier weakness that cause bAVM instability.

Diverse studies have noted the presence of immune cells in and around malformed vessels in experimental hereditary hemorrhagic telangiectasia (HHT) mouse models.^66–68^ A recent study showed that the depletion of MG/Mφ using PLX5622 led to a reduction in vascular integrity in the brains of Alk1-mutated HHT mice.^25^ These findings strongly support the hypothesis that activated MG/Mφ are involved in producing bAVM instability. However, the mechanisms by which malformed vessels recruit MG/Mφ to produce vascular weakening and BBB instability in mutant-KRAS-induced bAVMs are unknown. To investigate these mechanisms, we utilized our novel bAVM mouse model, which is based on the overexpression of mutant KRAS (KRAS^G12V^) selectively in the brain vascular ECs. Using this model, we now demonstrate that the MG/Mφ are densely present at the bAVM site and that both EC and MG/Mφ at bAVM produce high levels of pro-inflammatory IL-6 and IL-1β. To investigate the potential relevance of these findings, we used an in vitro BBB co-culture system containing KRAS-G12V-EC versus GFP-EC (control) to which we added Mφ to mimic the infiltration of Mφ into the bAVM location. As anticipated, the inclusion of Mφ in the BBB co-culture resulted in an accelerated loss of EC barrier integrity, as measured using the TEER assay. The enhanced loss of EC barrier integrity by Mφ is further supported by an in vitro BBB leakage test, which shows increased permeability to FITC-dextran and increased CAV1, an indicator of increased transcytosis. Interestingly, although the reduced TEER ratio is maintained by KRAS-G12V-EC at 3 days post-transfection, the permeability of FITC-dextran is unchanged. The data suggest that reduced TEER, as an indicator of compromised BBB function, precedes significant functional BBB disruption, which ultimately enables blood leakage in vivo. Remarkably, the Mφ-enhanced BBB permeability is reversed by minocycline treatment in the KRAS-G12V-EC. The data indicate that anti-inflammatory strategies targeting the source of inflammation can maintain the integrity of the BBB and contribute to improving the stability of the bAVM.

To better understand the potential mechanisms associated with the loss of BBB integrity due to KRAS-G12V-EC mutation and MG/Mφ infiltration, we further investigated the interaction between KRAS-G12V-EC and MG/Mφ regarding the amplification of deleterious inflammation and EC junctional proteins. To achieve this objective, we analyzed the gene expression changes in KRAS-G12V-EC, conducted KRAS-G12V-EC-CM transfer onto MG, and used the 3D BBB co-culture system containing KRAS-G12V-EC in the presence of Mφ. We found that KRAS-G12V-EC versus GFP-EC (control) have elevated expression of pro-inflammatory genes, similar to those found in bAVMs, including IL-1β, IL-6, and MCP-1.^12^ We have also found that KRAS-G12V-EC have lower expression of several junctional proteins that mediate cell-to-cell and cell-to-matrix adhesion, potentially suggesting a reason for BBB instability. The pro-inflammatory phenotype of KRAS-G12V-EC suggest that these cells may actually initiate the activation of adjacent MG and attract infiltration of Mφ. In agreement with this notion, the CM from KRAS-G12V-EC transferred onto BV2-MG produced strong pro-inflammatory responses in MG, including the increased expression of IL-6, IL-1β, MMP9, and CSF1R genes, suggesting changes in MG proliferation (CSF1R) and an increase in MG propensity to break down the BBB (MMP9). Because BBB instability, due to reduced expression of junction proteins and potential breakdown, could underlie the pathogenesis of bAVM, we next applied a 3D BBB model to establish KRAS-EC interaction with Mφ. Using this model and analyzing the CM for its secretome composition, we further confirmed that KRAS-G12V-EC secrete high levels of pro-inflammatory factors, including IL-1β, MCP-1, MMP-9, and endoglin, which are known to adversely affect the production of tight junction constituents and cause increased BBB permeability.^69–73^ Furthermore, essential to our hypothesis on KRAS-EC enhancing pro-inflammatory phenotype of Mφ, we showed that KRAS-EC-CM induce the activation of Mφ, which in turn causes Mφ to secrete various inflammatory mediators, including MMP9, MMP3, C5a, and SDF-1, by analyzing the secretome in the abluminal chamber containing Mφ. Meanwhile, incomplete coverage of mural cells, including pericytes and vascular smooth muscle cells (VSMCs), is a hallmark of bAVM.^12,66,74^ Mural cells are a potential source of pro-inflammatory mediators, and disruption of pericyte/VSMC-Mφ interactions, driven by impaired chemokine (CCL2 and MIF) signaling, exacerbates inflammation.^75,76^ Therefore, an in vitro BBB system incorporating mural cells should be further assessed to test their potential to regulate an inflammatory environment together with KRAS-G12V-EC and Mφ.

MCP-1, which is elevated in KRAS-G12V-EC, is a key chemoattractant factor in Mφ recruitment, contributing to the rearrangement of tight junctions and promoting BBB instability.^77^ MCP-1 has also been shown to promote angiogenesis triggered by VEGF-A, thereby facilitating the formation of malformed vessels in bAVMs.^72,78^ A recent study found that soluble endoglin, which is expressed in KRAS-G12V-EC, can lead to angiogenesis and inflammation.^79^ We earlier confirmed that soluble endoglin stimulated the expression of inflammatory mediators, matrix metalloproteinases (MMP-2 and MMP-9), in addition to promoting aberrant vasculature development in bAVMs.^80^ Several studies showed that the activated complement factor C5a, which is robustly secreted by Mφ primed by KRAS-G12V-EC secretome, may induce smooth muscle contraction, blood vessel dilatation, immune cell recruitment, and enhancement of vascular permeability.^81^ C5a induces EC activation using the C5a receptor, resulting in enhanced expression of cell adhesion molecules, cytokines, and chemokines.^82^ Chemokine ligand (CCL) 28 is another factor secreted by Mφ primed by KRAS-G12V-EC secretome. CCL28 is known for promoting endothelial EC proliferation and migration, and overexpression of CCL28 promotes aberrant angiogenesis that can lead to vessel deformation.^83^ We believe that together, these chemokines secreted factors may contribute to EC malformation and increased vascular permeability and instability of malformed vessels in bAVMs, facilitating vessel rupture.

We seeded astrocytes to mimic the BBB structure and co-cultured with ECs and Mφ.^35,84^ While astrocytes were expected to form a well-built in vitro BBB, astrocytes can also increase inflammation in bAVM, as shown in Braf^V600E^-induced bAVM mice.^85^ Thus, astrocytes in our system may also contribute to inflammatory responses in KRAS-G12V-ECs or Mφ. Although our experiments showed that KRAS-EC-CM directly induces BV2-MG activation, even without astrocyte co-culture, further studies are needed to investigate how astrocytes contribute to bAVM instability. Additionally, the selectivity of the BR1 capsid, which predominantly transduces KRAS^G12V^ into the brain ECs, minimizes the direct effect of mutant KRAS on other cell types. However, the possibility of mutant KRAS- induced inflammatory responses on Mφ or MG that can directly trigger inflammation warrants further consideration.

Minocycline was shown to reduce inflammatory responses in various experimental neurological disease rodent models, including hemorrhagic stroke, Parkinson’s disease, and Alzheimer’s disease.^86,87^ Furthermore, minocycline has been tested in human bAVM patients and shown to be safe, which supports the conduct of larger clinical trials.^88^ Besides, minocycline has not yet been evaluated in experimental bAVM animal models caused by mutant KRAS. Thus, we utilized minocycline and demonstrated that inflammation amplified through KRAS-G12V-ECs:MG/Mφ crosstalk is inhibited by minocycline, further suggesting that exacerbation of vascular pathology at bAVM is driven by inflammation. Our study suggests that further investigations into specific cellular targets, for example, EC, MG, and Mφ, involved in the minocycline effect are warranted.

**Figure 7.**
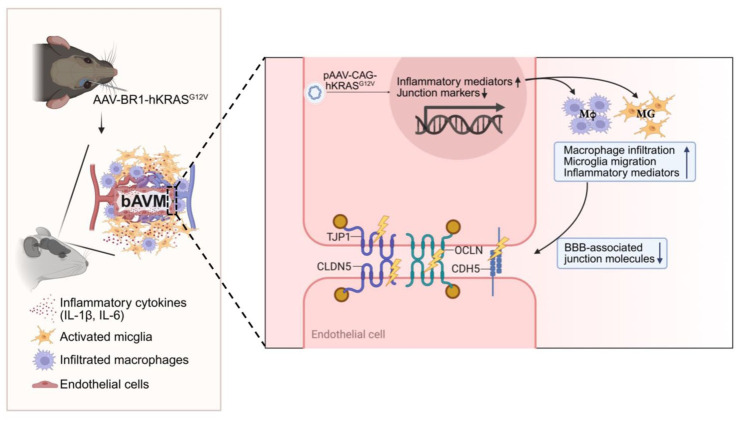
KRAS-G12V-EC collaborate with MG/Mφ in increasing bAVM instability. Overexpression of mutant KRAS (KRAS^G12V^) in EC is sufficient to cause the development of bAVM. Surrounding bAVM displays a dense presence of MG/Mφ, which induce the production of inflammatory cytokines, including IL-6 and IL-1β. The mutant KRAS drove the production of inflammatory mediators in EC while reducing the expression of EC junction markers. Mutant KRAS compromised EC barrier integrity, which was worsened by Mφ. Overall, MG/Mφ collaborate with KRAS-G12V-EC to weaken vascular integrity, causing the instability of malformed vessels in bAVM. IL-1β, interleukin-1β; IL-6, interleukin-6; TJP1, tight junction protein 1; OCLN, occludin; CLDN5, claudin-5; CDH5, VE-cadherin (cadherin-5); BBB, blood-brain barrier; MG, microglia; Mφ, macrophages.

Taken together, the results presented here suggest that KRAS mutations in ECs induce pro-inflammatory EC activation, leading to MG/Mφ activation and recruitment, which ultimately compromise vascular integrity in bAVMs (Figure 7). Our findings also suggest that targeting this vascular inflammation is a worthwhile area of further investigation into pharmacological therapeutics for bAVMs.

## Supplemental Material

sj-docx-1-jcb-10.1177_0271678X261421424 – Supplemental material for Mutant KRAS in brain endothelial cells promotes vascular inflammation and impairs vascular integrity in brain arteriovenous malformation Supplemental material, sj-docx-1-jcb-10.1177_0271678X261421424 for Mutant KRAS in brain endothelial cells promotes vascular inflammation and impairs vascular integrity in brain arteriovenous malformation by Jung-Eun Park, Bridger H Freeman, Hyejin Park, Sehee Kim, Adrian E Bafor, Ohnmar Myint, Song Gao, Zhen Xu, Jakob Körbelin, Jaroslaw Aronowski, Peng Roc Chen, Eunhee Kim and Eun S Park in Journal of Cerebral Blood Flow & Metabolism
