# Supplementary material for: Mutant KRAS in brain endothelial cells promotes vascular inflammation and impairs vascular integrity in brain arteriovenous malformation
Source: J Cereb Blood Flow Metab. 2026 Feb 16:0271678X261421424. Online ahead of print. doi: 10.1177/0271678X261421424 (PMC12913046; doi:10.1177/0271678X261421424)
Supplement: sj-docx-1-jcb-10.1177_0271678X261421424 – Supplemental material for Mutant KRAS in brain endothelial cells promotes vascular inflammation and impairs vascular integrity in brain arteriovenous malformation [file sj-docx-1-jcb-10.1177_0271678X261421424.docx]

**SUPPLEMENTAL FILES**

**Mutant KRAS in endothelial cells promotes vascular inflammation and impairs vascular integrity in brain arteriovenous malformation**

Jung-Eun Park^1^, Bridger H. Freeman^1^, Hyejin Park^1^, Sehee Kim^1^, Adrian E. Bafor^1^, Ohnmar Myint^1^, Song Gao^1^, Zhen Xu^1^, Jakob Körbelin^2^, Jaroslaw Aronowski^3,4^, Peng Roc Chen^1^, Eunhee Kim^1^, Eun S. Park^1,4*^

^1^Vivian L. Smith Department of Neurosurgery, McGovern Medical School, The University of Texas Health Science Center at Houston, Houston, TX 77030, USA.

^2^Department of Oncology, Hematology and Bone Marrow Transplantation, University Medical Center Hamburg-Eppendorf, 20246 Hamburg, Germany.

^3^Department of Neurology, McGovern Medical School, The University of Texas Health Science Center at Houston, Houston, TX 77030, USA.

^4^Center for Neuroimmunology and Glial Biology, The Brown Foundation Institute of Molecular Medicine, The University of Texas Health Science Center at Houston, Houston, TX 77030, USA.

^*^Corresponding author: Eun S. Park (E.S.P.), Email: [Eunsu.park@uth.tmc.edu](mailto:Eunsu.park@uth.tmc.edu)

**
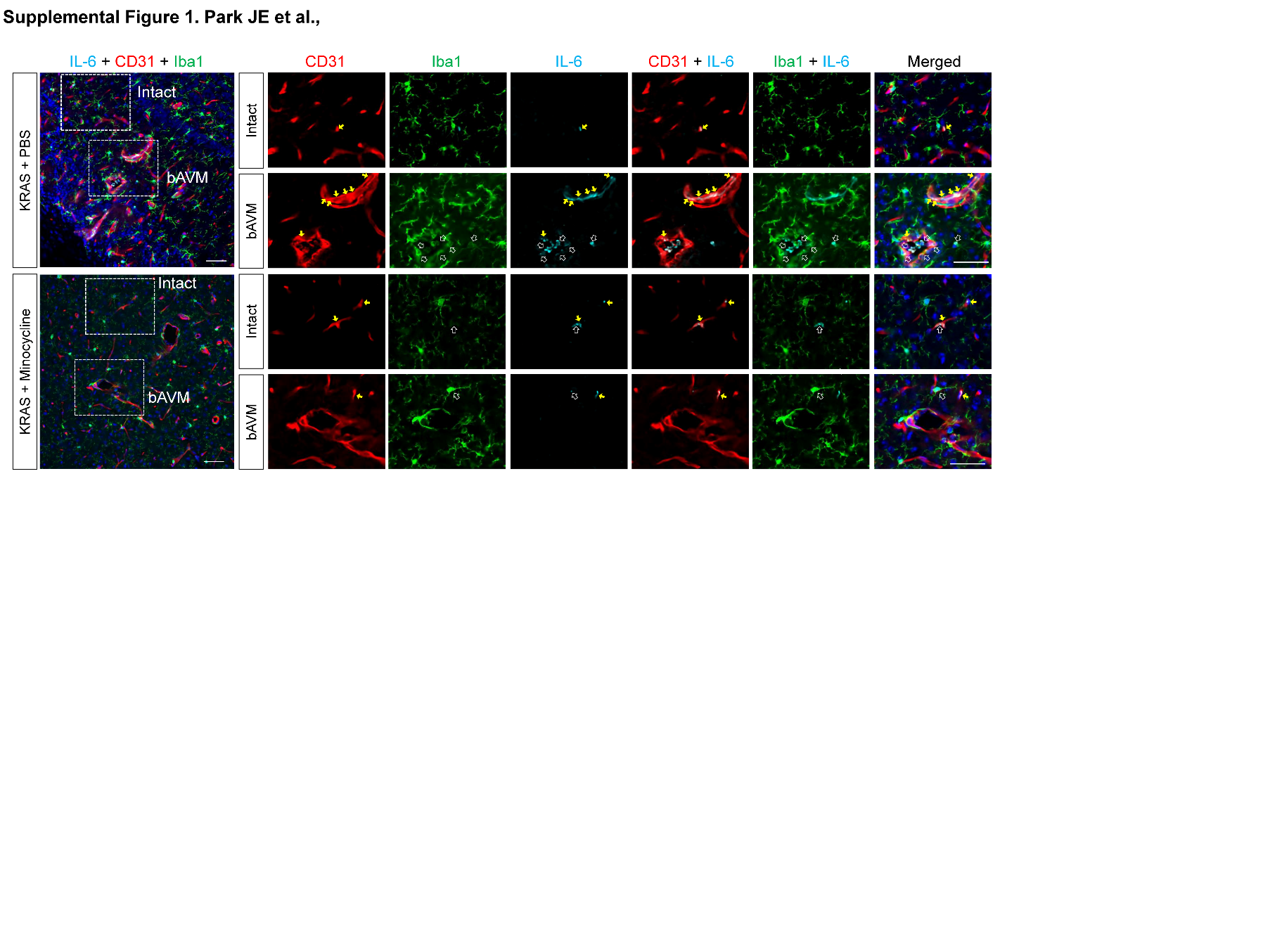
**

**Supplemental Figure 1. Treatment of KRAS^G12V/bEC^ mice with minocycline reduces IL-6 expression on the EC and MG/Mϕ.** Representative low-power images show the intact and bAVM area in KRAS^G12V/bEC^ mouse. Single-channel image of immunofluorescence showing the expression of IL-6 on the CD31^+^ and Iba1^+^ cells by AAV-BR1-KRAS^G12V^ injection with or without treatment with minocycline; The yellow arrow indicates expression of IL-6 in CD31^+^ vessels, while the white empty arrow indicates expression of IL-6 in Iba1^+^ MG/Mϕ; Minocycline decreased IL-6 expression on the CD31^+^ and Iba1^+^ cells in bAVM area. Scale bar = 50 μm.

**
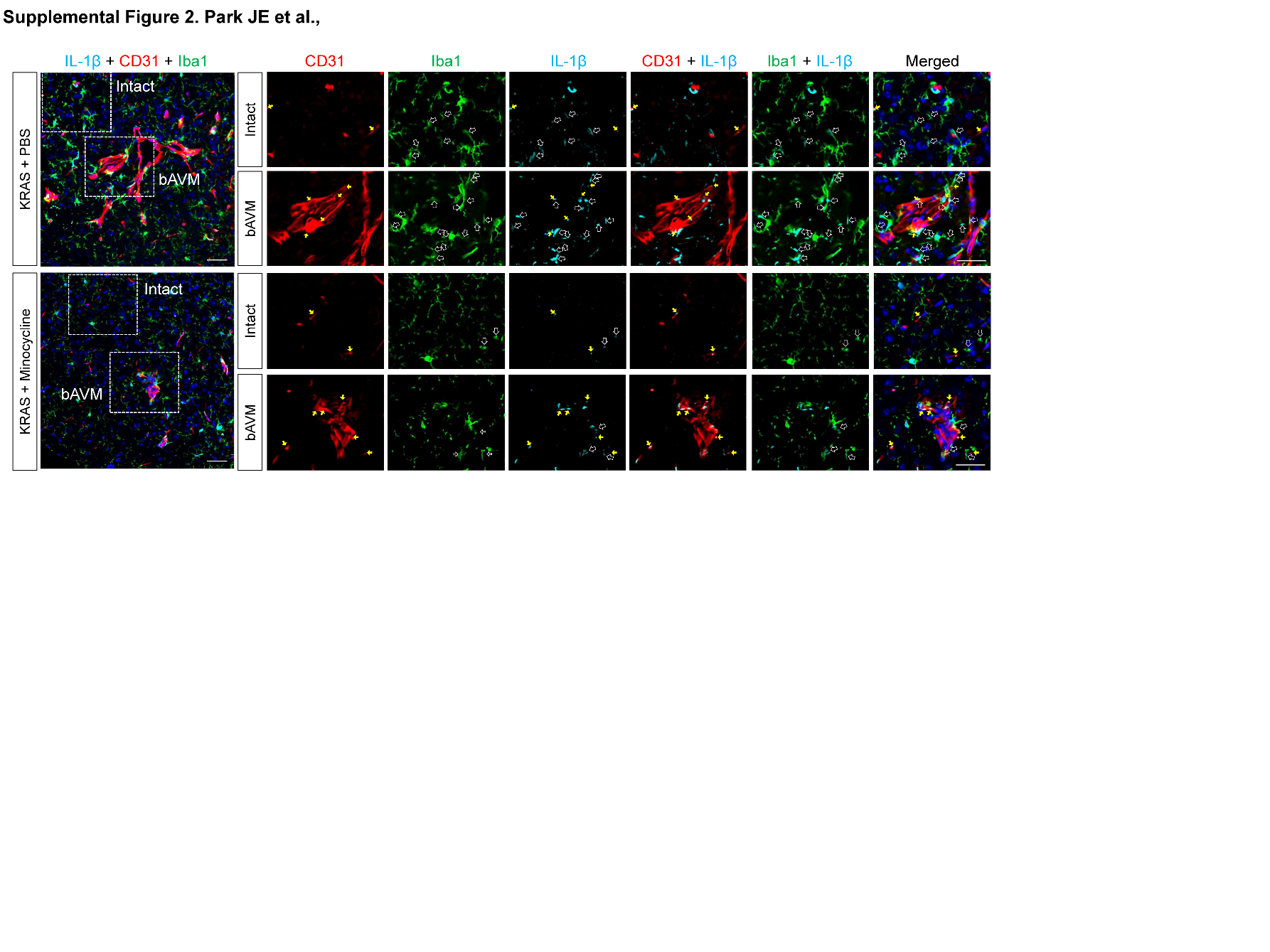
**

**Supplemental Figure 2. Treatment of KRAS^G12V/bEC^ mice with minocycline reduces IL-1β expression on the EC and MG/Mϕ.** Representative low-power images show the intact and bAVM area in KRAS^G12V/bEC^ mouse. Single-channel image of immunofluorescence showing the expression of IL-1β on the CD31^+^ and Iba1^+^ cells by AAV-BR1-KRAS^G12V^ injection with or without treatment with minocycline; The yellow arrow indicates expression of IL-1β in CD31^+^ vessels, while the white empty arrow indicates expression of IL-1β in Iba1^+^ MG/Mϕ; Minocycline decreased IL-1β expression on the CD31^+^ and Iba1^+^ cells in bAVM area. Scale bar = 50 μm.

**
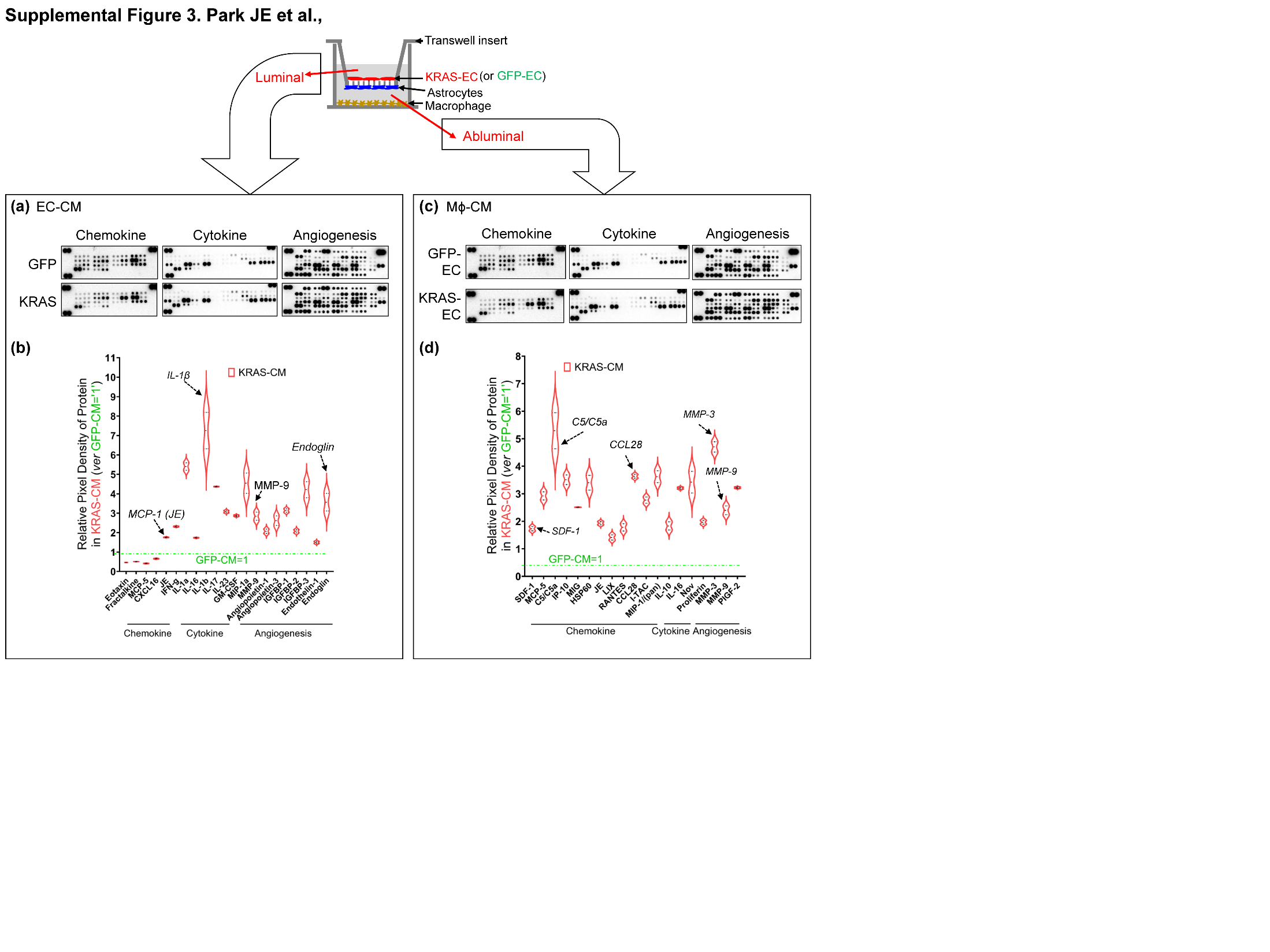
**

**Supplemental Figure 3. KRAS-G12V-EC and Mϕ release chemokines, inflammatory cytokines, and angiogenic factors in 3D BBB co-culture systems**. Dot blotting was performed with conditioned medium (CM) obtained from KRAS-G12V-EC (**a, b**) and Mϕ (**c, d**) in the 3D BBB co-culture system comprising KRAS-G12V-EC or GFP-EC at 3 days. (**a, c**) Representative Dot blot images showing the chemokines, cytokines, and angiogenic molecules secreted from EC (a) and Mϕ (c). Each protein is visualized by a duplicated spot; (**b, d**) The quantification showing the differential expression of soluble chemokine, cytokine, and angiogenesis-related molecules obtained using CM from EC (b) and Mϕ (d) The relative pixel density of each protein released from EC or Mϕ was measured using ImageJ, and the difference between KRAS-G12V-EC-CM and GFP-EC-CM was compared. The y-axis represents the relative intensity of each protein *versus* GFP-CM (standardized to 1). Representative chemokines, cytokines, and angiogenesis-related molecules are noted as increased in the CM from EC or Mϕ.

**
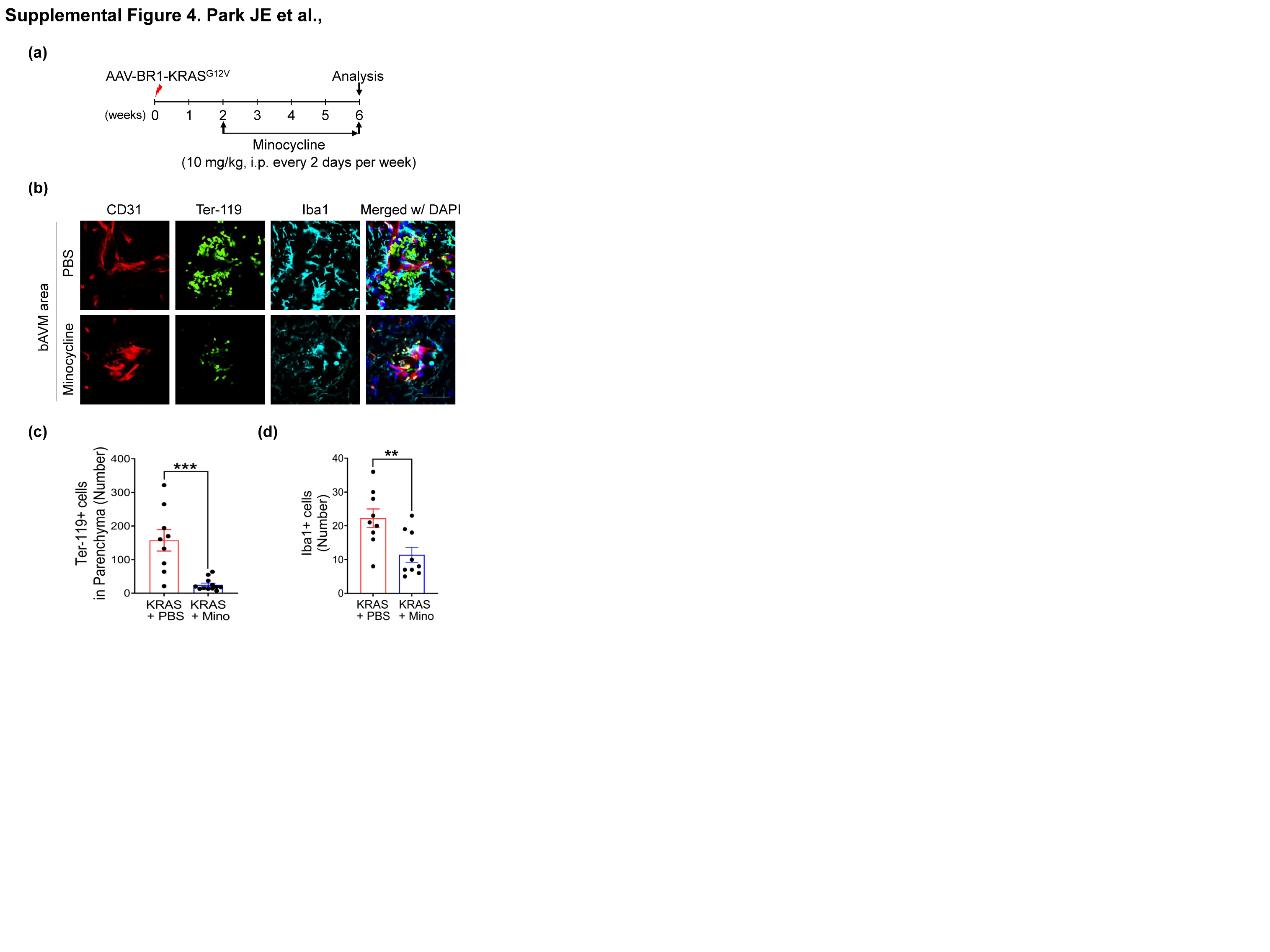
**

**Supplemental Figure 4. Minocycline treatment attenuates red blood cell leakage in KRAS^G12V/bEC^ mice.** (**a**) KRAS^G12V/bEC^ mice received minocycline daily (i.p.) starting 2 weeks post-AAV-BR1-KRAS^G12V^ injection for 4 weeks. (**b**) Ter-119 immunostaining displays the reduced ICH in minocycline-treated KRAS^G12V/bEC^ mice. (**c, d**) Quantification of Ter-119^+^ cells in parenchyma (c) and Iba1^+^ cells (d). A dot indicates each ROI (n=9-12) obtained from the mouse (n=4). Unpaired t-test. ***, *p*<0.001, Scale bar: 50 μm.
